# Muscle in Endocrinology: From Skeletal Muscle Hormone Regulation to Myokine Secretion and Its Implications in Endocrine–Metabolic Diseases

**DOI:** 10.3390/jcm14134490

**Published:** 2025-06-25

**Authors:** Pedro Iglesias

**Affiliations:** 1 Department of Endocrinology and Nutrition, Hospital Universitario Puerta de Hierro Majadahonda, Calle Joaquín Rodrigo, 1, Majadahonda, 28222 Madrid, Spain; piglo65@gmail.com; 2 Instituto de Investigación Sanitaria Puerta de Hierro Segovia de Arana, Majadahonda, 28222 Madrid, Spain

**Keywords:** endocrine–metabolic diseases, skeletal muscle, myokine, irisin, fibronectin type III domain-containing protein 5, interleukin-6, interleukin-15, brain-derived neurotrophic factor, myonectin, myostatin, meteorin-like, secreted protein acidic and rich in cysteine, leukemia inhibitory factor, fibroblast growth factor 21

## Abstract

Skeletal muscle, traditionally recognized for its motor function, has emerged as a key endocrine organ involved in metabolic regulation and interorgan communication. This narrative review addresses the dual role of muscle as a target tissue for classical hormones—such as growth hormone (GH), insulin-like growth factor type 1 (IGF-1), thyroid hormones, and sex steroids—and as a source of myokines, bioactive peptides released in response to muscle contraction that exert autocrine, paracrine, and endocrine effects. Several relevant myokines are discussed, such as irisin and Metrnl-like myokines (Metrnl), which mediate exercise-associated metabolic benefits, including improved insulin sensitivity, induction of thermogenesis in adipose tissue, and immunometabolic modulations. It also examines how muscle endocrine dysfunction, caused by chronic inflammation, hormone resistance, or sedentary lifestyle, contributes to the development and progression of metabolic diseases such as obesity, type 2 diabetes, and sarcopenia, highlighting the importance of muscle mass in the prognosis of these pathologies. Finally, the therapeutic potential of interventions aimed at preserving or enhancing muscle function—through physical exercise, hormone therapy and anabolic agents—is highlighted, together with the growing research on myokines as biomarkers and pharmacological targets. This review expands the understanding of muscle in endocrinology, proposing an integrative approach that recognizes its central role in metabolic health and its potential to innovate the clinical management of endocrine–metabolic diseases.

## 1. Introduction

**Figure 1:**
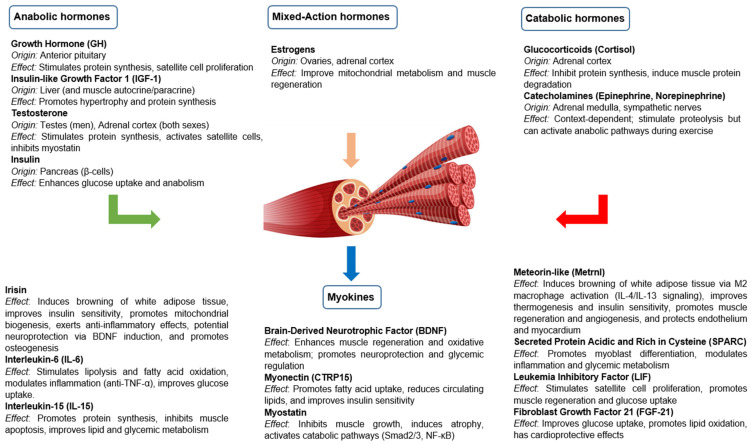
Hormonal regulation and myokine secretion of skeletal muscle under physiological conditions.

For many years, skeletal muscle has been considered an organ with purely mechanical functions, responsible for generating force and facilitating movement. In addition to its contractile role, skeletal muscle is now recognized as a hormonally responsive and secretory organ. It interacts with classical endocrine signals and produces bioactive molecules with systemic effects [1,2,3,4,5]. This hormonal modulation is essential to preserve muscle mass and function, especially in physiological and pathological contexts where the anabolic–catabolic balance is disturbed. In addition to behaving as a target tissue for different hormones, skeletal muscle is now recognized as a secretory organ capable of releasing a variety of peptides and proteins called myokines [6,7,8]. Myokines are peptides produced by skeletal myocytes that act through autocrine, paracrine and endocrine mechanisms, participating in metabolic regulation, immune response and inter-organ communication. They influence multiple tissues, such as the liver, pancreas, adipose tissue, brain, cardiovascular system and bone, and mediate the beneficial effects of exercise, such as improved insulin sensitivity and decreased inflammation (Figure 1).

Skeletal muscle endocrine dysfunction, resulting from factors such as hormonal resistance, chronic inflammation or physical inactivity, plays a key role in the development of metabolic disorders such as obesity, type 2 diabetes, insulin resistance and gonadal dysfunction syndromes. Likewise, sarcopenia—characterized by the loss of muscle mass and quality—is associated with a worse prognosis in patients with complex endocrine diseases. In various metabolic diseases, alterations are observed in several myokines, such as interleukin-6 (IL-6), interleukin-15 (IL-15), metronin-like (Metrnl), irisin and leukemia inhibitory factor (LIF), whose levels and functions are modified, affecting energy metabolism, inflammation and interorgan communication. These myokinetic disruptions play a key role in the pathophysiology of these diseases and represent potential targets for the development of biomarkers and therapeutic strategies. Further understanding of the bidirectional interactions between muscle and the endocrine system not only improves the understanding of these clinical pictures, but also offers therapeutic opportunities focused on optimizing muscle function.

The recognition of skeletal muscle as an endocrine organ raises important clinical implications. Interventions aimed at preserving or improving muscle function—such as structured physical exercise, individualized hormonal therapy or the use of anabolic agents—could become effective therapeutic strategies to prevent and treat endocrine–metabolic disorders. It has been suggested that myokines might act directly on the brain parenchyma via crossing the blood–brain barrier [9,10]. Moreover, a bidirectional interaction between muscle and intestinal microbiota has been described, whose modulation influences muscle health, metabolism and inflammatory response, with therapeutic implications in aging and metabolic diseases [11]. In addition, myokines emerge as potential diagnostic biomarkers and pharmacological targets in the management of these pathologies. The integration of muscle biology in the clinical endocrinological approach represents, therefore, a paradigm shift with significant translational potential. In current clinical practice, this evidence reinforces the need to review the role of muscle in endocrinology, both as a hormonal target and as a source of signals with possible relevant therapeutic implications.

## 2. Methods

To ensure a comprehensive and structured overview of the topic, a search of the literature was conducted using relevant Medical Subject Headings (MeSH) terms such as “skeletal muscle”, “myokine”, “anabolic hormones”, “catabolic hormones”, “irisin”, “fibronectin type III domain-containing protein 5”, “interleukin-6”, “interleukin-15”, “brain-derived neurotrophic factor”, “myonectin”, “myostatin”, “meteorin-like”, “secreted protein acidic and rich in cysteine”, “leukemia inhibitory factor”, and “fibroblast growth factor 21”. Searches were performed in PubMed/Medline, Cochrane Database of Systematic Reviews, and Embase. References were selected based on their relevance, scientific quality, and contribution to the current understanding of the endocrine functions of skeletal muscle, with preference given to articles published in the last 5–10 years. Classical studies were also included when necessary to provide historical and mechanistic context.

## 3. Muscle as an Endocrine Target Organ

Skeletal muscle is not only a contractile tissue but also a dynamic endocrine target organ, responding to a variety of hormonal signals that regulate its growth, metabolism and function. Skeletal muscle homeostasis and functionality depend on a balance between anabolic and catabolic hormonal signals, which vary according to nutritional status, physical activity, age and pathophysiological environment. Table 1 summarizes the anabolic and catabolic effects of key hormones on skeletal muscle under physiological conditions, reflecting their typical roles in muscle metabolism, growth, and function.

### 3.1. Anabolic Hormones

Anabolic hormones play a central role in promoting muscle growth and maintenance by stimulating protein synthesis, inhibiting proteolysis and modulating muscle stem cell activity. These include GH, IGF-1, testosterone and insulin.

#### 3.1.1. GH/IGF-1

GH and IGF-1 are key hormones for skeletal muscle development and function, acting in a complementary manner and through both dependent and independent mechanisms.

GH promotes skeletal muscle growth and function by inducing IGF-1 synthesis in liver and peripheral tissues. GH and IGF-1 modulate myokine production and function, with IGF-1 being one of the major myokines with anabolic and regulatory effects on skeletal muscle, where IGF-1 acts as a key myokine that promotes muscle satellite cell proliferation and differentiation, protein synthesis and muscle hypertrophy [29]. In addition, by exerting a lipolytic effect, it favors the preservation of muscle mass in catabolic conditions [12,13,14].

IGF-1 is a central mediator of the anabolic effects of GH in skeletal muscle, acting mainly through the phosphatidylinositol 3-kinase (PI3K)/protein kinase B (Akt)/mammalian target of the rapamycin (mTOR) pathway to stimulate protein synthesis and reduce protein degradation, promoting muscle hypertrophy. It also promotes the proliferation and differentiation of satellite cells, essential for muscle regeneration and growth, and participates in the regulation of mitochondrial biogenesis and mitophagy during myogenic differentiation, thus contributing to mitochondrial remodeling and function [29,30,31,32].

GH deficiency is associated with reduced muscle mass and strength, due to reduced protein synthesis and satellite cell proliferation [33]. In children and adolescents, it compromises muscle development and may limit peak muscle mass [34]. In adults, it results in loss of lean mass, increased fat mass and decreased functional capacity, as well as possible metabolic alterations such as insulin resistance [35]. GH replacement therapy has been shown to improve these parameters, favoring body composition and muscle function [36].

On the other hand, situations of GH and IGF-1 excess such as acromegaly and gigantism are associated with an increase in muscle mass due to the anabolic effect of GH and IGF-1 excess; however, this increase does not always translate into a functional improvement [37,38]. Muscle quality may be compromised by intramuscular fatty infiltration, decreased stiffness and a state of high protein turnover, contributing to proximal muscle weakness and fatigue [39,40]. After treatment and hormonal normalization, a reduction in muscle mass and an increase in fat mass may be observed [41].

#### 3.1.2. Testosterone

Testosterone, the principal androgen, is a steroid hormone produced predominantly in the testes in males, and to a lesser extent in the ovaries in females. It is essential for the development and maintenance of male reproductive tissues, sexual function, bone and muscle health, and general well-being in both sexes. Among its many physiological functions, it plays an important role in the maintenance of skeletal muscle mass and function [42]. Several studies have confirmed that serum total testosterone levels are positively associated with muscle mass in adult males between 20 and 59 years of age but not with muscle strength [43]. Sex differences also play a role: in women, testosterone levels are considerably lower and no significant association with muscle mass or strength has been observed, possibly because of insufficient levels to induce anabolic effects or because of the greater influence of other hormonal factors [44,45]. In summary, clinical and experimental evidence supports the central role of testosterone as a modulator of muscle mass, with additive effects when combined with resistance exercise. However, its impact on strength and function should be interpreted with caution, especially in aging populations, where sarcopenia may be determined by multiple factors in addition to the hormonal environment.

The anabolic action of testosterone is manifested mainly through the stimulation of muscle protein synthesis and the inhibition of its degradation, thus promoting muscle hypertrophy. This effect is mediated by multiple cellular mechanisms, such as activation of satellite cells, increased expression of growth factors (e.g., IGF-1 and fibroblast growth factor 2, FGF2), and inhibition of myostatin, a negative regulator of muscle growth [46,47,48]. In addition, testosterone modulates the differentiation of pluripotent stem cells into muscle lineages, preventing their conversion into adipose tissue, which contributes to increased lean mass [49].

Hypogonadism, characterized by testosterone deficiency, is associated with a significant reduction in lean muscle mass and, to a lesser degree, muscle strength. This hormone deficiency compromises protein synthesis, promotes muscle degradation and alters the activation of satellite cells and the expression of anabolic factors such as IGF-1 and FGF2. Clinically, hypogonadal men present lower total and appendicular muscle mass, a higher proportion of body fat and a higher risk of falls and functional deterioration, especially with aging [47,50]. Although loss of strength is not always proportional to loss of mass, due to neuromuscular factors, testosterone replacement therapy (TRT) has been shown to improve body composition, increase fat-free mass and, to a lesser extent, muscle strength. These effects are enhanced when treatment is combined with resistance exercise or rehabilitation programs [47,50,51].

In bodybuilders and healthy individuals, the administration of testosterone or anabolic–androgenic steroids at supratherapeutic doses produces potent effects on skeletal muscle [50,52]. These compounds promote a marked increase in lean muscle mass and strength by stimulating protein synthesis, activating satellite cells and inhibiting protein degradation. Unlike hormone replacement therapy in hypogonadism, which seeks to restore physiological levels, use in bodybuilding involves very high concentrations, far exceeding normal values. Although these anabolic effects are evident, non-medical use carries significant risks, including cardiovascular, hepatic, endocrine, psychiatric and reproductive disorders [53]. In addition, prolonged abuse may suppress the hypothalamic–pituitary–gonadal axis, leading to secondary hypogonadism upon the discontinuation of treatment. Despite its ergogenic effects, its use is prohibited in competitive sport and lacks medical justification in eugonadal subjects.

#### 3.1.3. Insulin

Insulin plays a central role in skeletal muscle by coordinately regulating carbohydrate, lipid and protein metabolism and improving muscle perfusion [54,55]. It facilitates glucose uptake by translocation of the GLUT4 transporter, stimulates glycolysis and glycogen synthesis, and inhibits glycogen degradation [56]. In lipid metabolism, it reduces lipolysis in adipose tissue, decreases circulating fatty acids and inhibits lipid oxidation in muscle, favoring the use of glucose as an energy source [54]. At the protein level, it increases the synthesis and reduces the degradation of muscle proteins, promoting a positive nitrogen balance, especially in the presence of amino acids. These anabolic actions are mediated by the PI3K/Akt/mTOR pathway [57,58]. In addition, insulin increases postprandial muscle blood flow, facilitating nutrient delivery [59]. Insulin negatively regulates proinflammatory myokine secretion at rest, promotes an anabolic profile and, under conditions of insulin resistance, myokine production and function is altered, favoring inflammation and metabolic dysfunction [8]. Taken together, these effects are essential for maintaining metabolic homeostasis and functional integrity of the muscle.

Insulin resistance in skeletal muscle is a central element in the pathophysiology of type 2 diabetes and metabolic syndrome, compromising key functions such as glucose uptake, protein synthesis and muscle perfusion [60]. In insulin-resistant states, translocation of the GLUT4 transporter to the cell membrane is altered, compromising adequate postprandial glucose uptake and contributing to the development of hyperglycemia [61]. This defect is compounded by alterations in intracellular signaling (PI3K/Akt/mTOR), mitochondrial dysfunction and intramyocellular lipid accumulation, which interfere with insulin action through the activation of serine kinases [62]. In addition, chronic inflammation and reduced microvascular perfusion further limit glucose and insulin delivery to muscle [59]. All these mechanisms affect both energy metabolism and muscle mass and function, which favors the development of sarcopenia and contributes to a progressive worsening of metabolic deterioration.

Endogenous hyperinsulinism, as occurs in insulinomas or some forms of congenital hyperinsulinism, can have dual effects on skeletal muscle. In initial phases, hyperinsulinemia promotes glucose uptake, stimulates protein synthesis and inhibits proteolysis, with an anabolic effect mediated by the PI3K/Akt/mTOR pathway. However, prolonged exposure can induce muscle insulinoresistance and impair energy metabolism. In addition, recurrent hypoglycemia characteristic of this condition promotes a catabolic state with protein degradation, loss of muscle mass, fatigue and weakness [63,64,65].

### 3.2. Catabolic Hormones and Other Catabolic Mediators

Different hormones and catabolic mediators play a key role in muscle protein degradation, inhibition of protein synthesis and loss of muscle mass in various physiological and pathological conditions. These include glucocorticoids, catecholamines, myostatin and different proinflammatory cytokines. Their action is mainly exerted by activation of proteolytic pathways and inhibition of anabolic pathways, leading to a net loss of muscle mass and function.

#### 3.2.1. Glucocorticoids

Glucocorticoids induce muscle atrophy through multiple catabolic mechanisms that negatively affect skeletal muscle mass and function [15,16]. Glucocorticoids increase the expression of catabolic myokines (such as myostatin), decrease the expression and function of anabolic myokines (such as IGF-1), and induce IGF-1 resistance, promoting muscle atrophy and dysfunction [66,67]. They suppress protein synthesis by inhibiting the mTOR pathway and reducing IGF-1 signaling, as well as inducing insulin resistance and IGF-1 resistance [15,68,69]. In parallel, they activate protein degradation pathways such as the ubiquitin–proteasome system and the autophagy-lysosome, and increase the expression of factors such as myostatin, REDD1 (Regulated in Development and DNA Damage Response 1), KLF15 (Kruppel-like Factor 15) and GSK-3β (Glycogen Synthase Kinase-3 beta), which reinforce the blockade of protein synthesis and myogenic differentiation [15,16,67]. These effects translate clinically into a glucocorticoid myopathy, characterized by progressive proximal muscle weakness, which may be reversible with withdrawal of treatment and appropriate rehabilitation.

Glucocorticoid excess, endogenous or induced by prolonged treatment with prednisone (≥10–20 mg/day), causes a myopathy characterized by progressive, symmetrical and painless weakness, mainly involving the proximal musculature of the pelvic and shoulder girdles. As mentioned above, this condition is associated with loss of muscle mass, insulin and IGF-1 resistance and disruption of the mechanisms that regulate protein balance, with a predominance of catabolism. In contrast to inflammatory myopathies, creatine kinase is usually maintained within normal ranges [16,70].

Chronic hypocortisolism affects skeletal muscle with clinical manifestations of asthenia, nonspecific weakness and fatigue, especially in the lower extremities, without significant pain or atrophy [71]. These symptoms are related to decreased energy metabolism, due to decreased gluconeogenesis and glucose availability, especially during exercise or fasting. In primary adrenal insufficiency, loss of aldosterone can cause hyponatremia and hyperkalemia, altering neuromuscular excitability. At the molecular level, cortisol deficiency reduces the activation of catabolic pathways such as the ubiquitin–proteasome system, decreasing protein degradation. In addition, it increases insulin sensitivity and decreases urea excretion, reflecting less proteolysis. However, reduced basal energy expenditure and anorexia contribute to fatigue, weight loss and low tolerance to exertion [71,72].

#### 3.2.2. Catecholamines

Catecholamines, such as adrenaline and noradrenaline, act primarily as catabolic mediators of skeletal muscle, although they can induce transient anabolic responses depending on the hormonal and metabolic environment [5,17,18]. Catecholamines stimulate the production and release of myokines in skeletal muscle, facilitating their autocrine, paracrine and endocrine action, and contributing to the systemic effects of exercise on metabolism and homeostasis [7].

In states of stress, fasting or hypercatabolism (such as sepsis, trauma or chronic diseases), their catabolic effects predominate: they stimulate proteolysis through the activation of β-adrenergic receptors, which enhances pathways such as NF-κB and ubiquitin–proteasome, increases IL-6 production and favors the loss of muscle mass [17,73,74]. In addition, glucocorticoids can amplify this response by inducing myostatin expression and increasing catecholamine release [69,75]. However, in certain contexts such as intense exercise, catecholamines can also exert anabolic effects through activation of the cAMP/PKA and PI3K/Akt pathways, which promote protein synthesis and inhibit the nuclear translocation of Foxo3a, reducing proteolysis. They also modulate muscle energy metabolism by promoting the mobilization of glycogen and intramuscular lipids [18,76].

In pheochromocytoma, chronic hypersecretion of catecholamines produces a sustained hypermetabolic and catabolic state, with multiple repercussions on skeletal muscle. This hormonal excess increases resting energy expenditure (REE), promotes protein degradation and autophagy, and favors the mobilization of energy substrates via anaerobic glycolysis and lipolysis. As a result, patients may present loss of lean mass, muscle atrophy—particularly of type II fibers—early fatigue, and, in some cases, a clinical myopathy with proximal muscle weakness and myalgias. In addition, this condition is associated with an elevated inflammatory profile, with increased cytokines such as tumor necrosis factor-alpha (TNF-α), interleukin-6 (IL-6), and interleukin-8 (IL-8). After adrenalectomy, a normalization of REE, an increase in body mass index as well as body fat content, and a significant reduction in inflammatory markers are observed, supporting the functional and reversible nature of these catecholamine-induced muscle changes [77,78].

#### 3.2.3. Inflammatory Cytokines

Proinflammatory cytokines, such as TNF-α, IL-6 and IL-1β, play a dual role in muscle physiology: they are essential for myogenesis and the balance between anabolism and catabolism under normal conditions, but their chronic or excessive expression, as occurs in chronic inflammatory diseases (rheumatoid arthritis, COPD, cachexia, sarcopenia), promotes catabolic pathways that induce muscle atrophy [79,80,81,82,83]. These cytokines activate calpains, E3 ligases (atrogin-1, MuRF1) and NF-κB, facilitating protein degradation through the ubiquitin–proteasome system, and inhibit IGF-1/Akt-mediated anabolic signaling, favoring the activation of FoxO [84,85,86]. In addition, stimuli such as tissue damage or pathogen-associated molecules perpetuate local inflammation by activating the innate immune response, exacerbating the loss of muscle mass and function. Despite advances in understanding these molecular mechanisms, there are currently no FDA-approved drugs that can effectively treat these muscle wasting conditions [79].

### 3.3. Other Relevant Hormones

Skeletal muscle is also regulated by various hormones that influence its metabolism, growth, repair and function.

#### 3.3.1. Estrogens

Estrogens play a critical role in the regulation of skeletal muscle mass, strength and function, acting through mechanisms that include stimulation of muscle satellite cells, stabilization of cell membranes and enhancement of mitochondrial energy metabolism [19,20,21,22]. This influence on mitochondrial function is exerted, in part, through the regulation of genes involved in mitochondrial biogenesis, an effect mediated mainly by estrogen receptors ERα and ERβ. In parallel, estrogen-related receptors (ERRα and ERRγ), transcription factors closely linked to but distinct from the classical estrogen receptors, have been shown to be essential in both the basal and adaptive control of mitochondrial bioenergetics in the muscle [19,87].

Skeletal muscle weakness associated with aging is aggravated in women by estrogen deficiency following ovarian failure. This deficiency contributes to the loss of muscle strength both through reduced muscle mass and deterioration in the quality of the remaining tissue. Estrogen deficiency has been observed to promote apoptosis in skeletal muscle and to alter key processes such as myosin phosphorylation and satellite cell function, compromising the ability to generate force [88,89]. Some studies suggest benefits of estrogen therapy on muscle mass and strength, but the findings are contradictory, possibly due to methodological variations [89]. Finally, physical exercise contributes in a complementary way, amplifying the positive effects of estrogens on muscle function [21].

#### 3.3.2. Adipokines

Adipokines, especially leptin and adiponectin, play a key role in the communication between adipose tissue and skeletal muscle, regulating key processes such as energy metabolism, insulin sensitivity, inflammation and the maintenance of muscle mass and function [23,24,25]. Leptin promotes lipid oxidation and appetite regulation, and is essential for preserving muscle mass, although elevated levels may be associated with muscle dysfunction, especially in the context of obesity [24,90]. Moreover, leptin enhances the intracellular activation of thyroid hormones in muscle, which increases energy expenditure and may influence metabolic balance, particularly in the context of overweight and obesity [91]. Adiponectin, on the other hand, has anti-inflammatory and antioxidant effects, improves insulin sensitivity and protects against muscle atrophy through the activation of pathways such as AMPK and PGC-1α [90,92]. Other adipokines such as resistin, TNF-α and IL-6 contribute to an inflammatory environment that can aggravate muscle wasting [79,80,81,82,83,93].

#### 3.3.3. Thyroid Hormones

The thyroid hormones, thyroxine (T4) and triiodothyronine (T3), are essential for the development and preservation of muscle tissue. These hormones stimulate the expression of genes encoding contractile proteins, such as myosin and actin, necessary for muscle contraction, as well as genes involved in mitochondrial biogenesis and in the regulation of energy metabolism and the oxidative capacity of muscle [26,27,28]. Hypothyroidism can be associated with weakness and myopathy [94], while hyperthyroidism can induce muscle atrophy as a consequence of increased protein catabolism, altered muscle fiber composition and decreased exercise capacity [95,96,97].

## 4. Muscle as an Active Endocrine Organ: Secretion of Myokines

**Figure 2:**
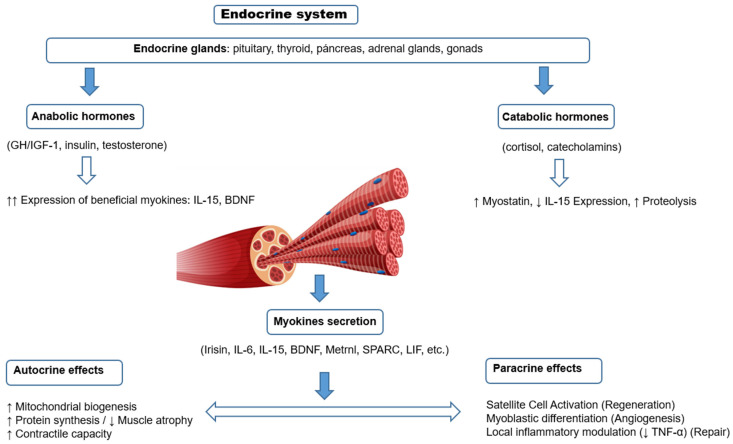
Scheme of local regulation of skeletal muscle structure and function by myokines (autocrine and paracrine effects), and their modulation by systemic hormones. Myokines are secreted in response to muscle contraction and exert local effects on muscle fibers (autocrine) or on neighboring cells such as satellite cells (paracrine). Their expression and function can be modulated positively by anabolic hormones (GH, IGF-1, insulin, testosterone) or negatively by catabolic hormones (glucocorticoids, catecholamines), generating a dynamic balance between muscle growth, repair or atrophy.

Myokines are bioactive proteins and peptides secreted by skeletal muscle in response to contraction, which exert their effects through autocrine, paracrine and endocrine mechanisms (Figure 2). These molecules act on multiple organs and tissues, participating in the regulation of energy metabolism, inflammatory response, cardiovascular health, neurological function and aging-related processes (Table 2). Over the past few years, numerous myokines with diverse functions have been identified, and the characterization of new molecules continues to expand this field of research. The following is an updated synthesis of the available knowledge on the main myokines described to date, with emphasis on their origin, mechanisms of action, physiological effects and clinical relevance.

Myokines display a dual functional profile that depends on the physiological or pathophysiological context in which they are secreted. Under physiological conditions, particularly during acute exercise, myokines such as muscle-derived IL-6, IL-15, Metrnl, and SPARC exert anti-inflammatory effects, reduce TNF-α, promote insulin sensitivity, and support metabolic homeostasis by enhancing immune regulation and metabolic flexibility. In contrast, pathophysiological states—including sedentary lifestyle, obesity, chronic inflammation, and glucocorticoid excess—favor a shift toward a proinflammatory and catabolic myokine profile, characterized by increased levels of myostatin, non-muscle-derived IL-6, and TNF-α. These myokines are associated with sarcopenia, insulin resistance, and immunometabolic dysregulation. This context-dependent behavior highlights the critical role of myokines in maintaining health and contributing to disease progression when homeostatic balance is disrupted [7,103,126,127,128].

The molecular mechanisms that regulate the expression and secretion of myokines involve various intracellular signaling pathways, among which the AMP-activated protein kinase (AMPK) pathway, signaling through nuclear factor kappa B (NF-κB) and pathways mediated by mitogen-activated protein kinases (MAPK) stand out. In addition, factors such as oxidative stress, insulin receptor activation and intracellular calcium dynamics modulate the synthesis and release of these molecules. These regulatory pathways allow for a precise adaptive response of skeletal muscle to different physiological and pathological stimuli, thus modulating the endocrine and paracrine role of myokines [8,129,130].

In myopathies, myokines regulate the tissue environment and can promote both muscle regeneration and damage, depending on the balance between inflammatory signals. Altered expression of myokines such as IL-6, IL-15, Irisin and myonectin has been observed. The chronic activation of pathways such as NF-κB or AMPK-PGC1α dysfunction contribute to the progression of damage. These alterations position myokines as potential biomarkers and therapeutic targets [126,131].

### 4.1. Irisin

Irisin is a myokine secreted mainly by skeletal muscle in response to physical exercise, especially aerobic exercise, through the proteolytic cleavage of the FNDC5 protein (Fibronectin Type III Domain-Containing Protein 5), whose expression is induced by the PGC-1α coactivator (Peroxisome proliferator-activated receptor gamma coactivator 1-alpha) [98,132,133]. Identified in 2012 by Boström et al. [134], this molecule acts as an interorganic messenger with multiple physiological effects. Its most outstanding action is the induction of browning of white adipose tissue, favoring conversion to thermogenic beige tissue, which increases energy expenditure and improves metabolic homeostasis. This mechanism is associated with increased insulin sensitivity, improved glucose uptake in muscle and adipose tissue, and a healthier metabolic profile, with therapeutic potential in obesity, type 2 diabetes and metabolic syndrome [98,99].

Moreover, irisin promotes mitochondrial biogenesis and muscle oxidative capacity, exerts anti-inflammatory effects and has shown implications in the central nervous system, where it could have a neuroprotective role through the induction of BDNF, with interest in diseases such as Alzheimer’s disease [99,132,133]. Positive effects on bone metabolism, including stimulation of osteogenesis, are also attributed to it [135]. Despite their potential, questions remain about their mechanisms of action and the standardization of their measurement in humans, as well as the validation of their receptors, among which αV/β5 integrins have been proposed [136]. Irisin therefore represents a key link between physical exercise and multiple systemic benefits, with potential therapeutic applications in metabolic, muscular and neurodegenerative diseases.

### 4.2. Fibronectin Type III Domain-Containing Protein 5

Fibronectin type III domain-containing protein 5 (FNDC5) is a transmembrane glycoprotein expressed mainly in skeletal muscle, heart, brain and liver and encoded by the FNDC5 gene. As discussed above, this protein acts as a precursor of irisin. In addition, FNDC5/irisin functionally interacts with Fibroblast Growth Factor 21 (FGF21), another metabolic regulator, and bioinformatic studies have identified polymorphisms in FNDC5 that could affect this interaction, influencing browning efficiency and susceptibility to metabolic disorders such as obesity and type 2 diabetes [137].

While most of the systemic effects attributed to FNDC5 are actually mediated by its cleaved form (irisin), recent research suggests that the full protein may have independent functions, especially at the local level in tissues such as brain and liver [138,139]. Therefore, FNDC5 seems to correspond to a multifunctional protein with therapeutic potential in metabolic and neurodegenerative diseases, although many aspects of its molecular and physiological biology still require further understanding.

### 4.3. Interleukin-6 of Muscle Origin

IL-6 of muscular origin is a myokine secreted by skeletal muscle fibers in response to contraction during exercise, especially prolonged and moderate or high intensity exercise. Unlike IL-6 of immune origin, its release is not associated with tissue damage or inflammatory processes but has regulatory and adaptive functions with anti-inflammatory and metabolic effects. It acts as a signaling molecule between muscle and liver, adipose tissue and other organs, promoting energy homeostasis during exercise. It stimulates lipolysis and fatty acid oxidation, increases hepatic gluconeogenesis and improves glucose uptake in muscle, helping to maintain glycemia [100,101].

In addition, muscle IL-6 modulates the immune response by inhibiting TNF-α production and stimulating anti-inflammatory cytokines such as IL-10 and IL-1ra, which favors the reduction of chronic low-grade inflammation observed in pathologies such as obesity and type 2 diabetes. It has also been linked to improvements in mitochondrial biogenesis and skeletal muscle energy efficiency, through pathways such as JAK/STAT (Janus kinase/signal transducer and activator of transcription). Its pleiotropic role includes possible effects on the central nervous system, where it could influence the regulation of appetite and energy expenditure [6,140]. Overall, muscle-derived IL-6 plays a central role in the metabolic adaptations induced by exercise and represents a potential therapeutic target for metabolic diseases.

### 4.4. Interleukin-15

Interleukin 15 (IL-15) is a pleiotropic cytokine that, in addition to its well-established immunological role, acts as a key myokine secreted by skeletal muscle, especially in response to physical exercise [141]. Its synthesis occurs mainly in muscle fibers, where IL-15 mRNA presents its highest levels, and its secretion is modulated by the intensity, duration and type of muscle contraction. IL-15 acts through a tricomponent receptor (IL-15Rα, IL-2Rβ and γc), with complex signaling that includes trans-presentation of the IL-15/IL-15Rα complex to other cells. This myokine promotes muscle mass growth and maintenance by stimulating contractile protein synthesis, inhibiting muscle fiber apoptosis, reducing intramuscular fat infiltration, and promoting mitochondrial biogenesis through the activation of PPARδ and PGC-1α. In addition, IL-15 has relevant effects on bone and lipid metabolism: it regulates osteoclastogenesis, reduces visceral fat, stimulates lipid oxidation and improves glucose uptake in a partially insulin-independent manner [102,103,104,105,106].

As a myokine, IL-15 plays a central role in the connection between exercise, metabolism and the immune system. Its positive metabolic action makes it a promising candidate for the treatment of pathologies such as visceral obesity, sarcopenia, type 2 diabetes and musculoskeletal degenerative diseases. In addition, its ability to activate NK cells and CD8^+^ T lymphocytes gives IL-15 potential in antitumor immunotherapy, with applications in the treatment of cancer and autoimmune diseases. Low IL-15 levels have been associated with sarcopenia, sarcopenic obesity and metabolic syndrome, which reinforces its value as a functional and therapeutic marker. Finally, its modulation by physical exercise represents a non-pharmacological strategy to improve the metabolic and structural health of the musculoskeletal system, while therapies based on IL-15 agonists or modulators are under investigation in the context of immunology and oncology [102,142].

### 4.5. Brain-Derived Neurotrophic Factor of Muscle Origin

Brain-derived neurotrophic factor (BDNF) is a neurotrophin essential for neuronal survival and plasticity, widely distributed in the central nervous system and, more recently, identified in peripheral tissues such as skeletal muscle [9,143]. Its main action is exerted through TrkB and p75NTR receptors, activating intracellular pathways such as MAPK, PI3K and PLC-γ, which are essential for neuronal differentiation, synaptic memory and the modulation of neurotransmitters such as serotonin and dopamine [144]. Beyond its neurological role, BDNF has emerged as a key regulator of energy metabolism, with effects on appetite, caloric expenditure and glycemic homeostasis, especially at the hypothalamic level [107,108].

From an endocrine perspective, low levels of BDNF are associated with obesity, insulin resistance and type 2 diabetes, possibly because of its role in pancreatic β-cell function and central regulation of glucose uptake. In addition, its expression is altered in conditions such as hypothalamic obesity and depression, with a clear link between metabolic and mental health [145,146]. In skeletal muscle, BDNF acts in an autocrine and paracrine manner, promoting regeneration, neuromuscular function and oxidative metabolism, increasing its expression after physical exercise. Interventions such as regular exercise, intermittent fasting and potentially GLP-1 agonists have shown the ability to raise its levels, positioning it as a metabolic biomarker and potential therapeutic target in endocrinology [143,147].

### 4.6. Myonectin

Myonectin, also known as C1q/TNF-related protein 15 (CTRP15) or erythroferrone, is a myokine secreted predominantly by skeletal muscle in response to metabolic stimuli such as exercise or nutrient intake [109]. It belongs to the C1q/TNF-related protein family and plays a key role in the regulation of lipid and glycemic metabolism. It promotes fatty acid uptake in liver and adipose tissue, reduces postprandial circulating free fatty acid levels and improves insulin sensitivity in liver and muscle. In addition, it can modulate hepatic gluconeogenesis and participate in metabolic pathways related to adiponectin, leptin and even hypothalamic signals associated with appetite [109,110,111,112].

Several studies show that aerobic exercise and high-intensity interval training (HIIT) increase myonectin levels in overweight or obese people, improving parameters such as HOMA-IR, fasting blood glucose and lipid profile [148,149]. In animal models, its overexpression improves insulin resistance, hepatic steatosis and muscle metabolism, also protecting against atrophy through activation of the AMPK/PGC1α pathway [112]. However, in healthy individuals, the evidence on the response to exercise is variable. It has been suggested that myonectin may regulate lipid metabolism and muscle mass after bariatric surgery [150]. In addition, myonectin is emerging as an early biomarker and potential therapeutic target in diseases such as type 2 diabetes, metabolic syndrome and NAFLD, although more robust clinical studies are required to define its systemic endocrine role and to optimize interventions based on its modulation.

### 4.7. Myostatin

Myostatin, also known as GDF-8 (Growth Differentiation Factor 8), is a regulatory protein belonging to the TGF-β (transforming growth factor beta) family. Its main function is to act as an inhibitor of skeletal muscle growth and regeneration, where it is predominantly produced and released. Following its synthesis in muscle, myostatin is secreted into the extracellular milieu and can act in an autocrine, paracrine and, to a lesser extent, endocrine manner [29,113].

Physiologically, myostatin limits muscle hypertrophy by suppressing the proliferation and differentiation of satellite cells and myoblasts. Its action is exerted through activation of the Smad2/3 pathway, which inhibits the Akt/mTORC1 axis, essential for protein synthesis. In addition, it can stimulate other catabolic pathways such as NF-κB and p38 MAPK, especially under conditions of oxidative stress, chronic inflammation or glucocorticoid exposure, which contributes to muscle atrophy [29,113]. Myostatin expression is increased in chronic inflammatory pathologies, such as COPD [16].

Genetic or pharmacological inhibition of myostatin produces a marked increase in muscle mass, as has been observed in animal models (e.g., “double-muscled” mice) and in human cases with inactivating mutations of the MSTN gene. In addition, administration of testosterone or anabolic steroids can reduce myostatin expression, contributing to its anabolic effects on muscle. Given its central role in the control of muscle growth, myostatin is currently being investigated as a possible therapeutic target in pathologies characterized by loss of muscle mass, such as sarcopenia, muscular dystrophy and cachexia [151,152,153,154,155].

### 4.8. Meteorin-like

Meteorin-like (Metrnl) is a secreted protein that acts as a myokine and adipokine, expressed in skeletal muscle, adipose tissue, macrophages and heart [114,156]. Its synthesis and secretion increase in response to physical exercise, especially endurance exercise, and are also modulated by metabolic states such as obesity, diabetes, fasting and caloric restriction. In muscle, Metrnl is induced by contraction and activated by intracellular pathways such as AMPK and PPARγ, participating in metabolic and immune signaling. It is associated with systemic effects on cardiovascular health, acting as a key exerkine linking physical activity with metabolic and vascular adaptations. Metrnl stimulates the browning of white adipose tissue through M2 macrophage activation and IL-4/IL-13 signaling, thereby improving thermogenesis, insulin sensitivity, and lipid oxidation [114,115]. It also enhances mitochondrial biogenesis, angiogenesis, and muscle regeneration after damage or inflammation, and has shown protective effects on endothelium, vascular remodeling, and myocardium after infarction [156].

Therapeutically, Metrnl represents a potential diagnostic and therapeutic tool in cardiovascular, metabolic, and muscular diseases. In the clinical context, its plasma levels have been associated with both acute cardiovascular events and chronic diseases such as heart failure and type 2 diabetes, reflecting its dual role as a protective mediator and stress biomarker. Preclinical studies suggest that it may improve post-infarction cardiac recovery, reduce the risk of atherosclerosis, and mitigate systemic inflammation. In addition, its ability to promote muscle regeneration and combat sarcopenia or diseases such as Duchenne muscular dystrophy positions it as an attractive therapeutic target. However, questions remain about the specificity of its receptors, its sustained regulation during chronic training, and its translation into human therapies. Even so, Metrnl is emerging as a molecular link between exercise and overall health, with great potential for the prevention and treatment of diseases linked to metabolism and the cardiovascular system [157,158,159,160].

### 4.9. Secreted Protein Acidic and Rich in Cysteine

Secreted protein acidic and rich in cysteine (SPARC), also known as osteonectin, is an exercise-induced myokine secreted by skeletal muscle and other tissues such as bone and adipose tissue [161]. Its synthesis increases in response to stimuli such as muscle contraction, tissue damage or mechanical stress, and its regulation is mediated by pathways such as AMPK and the STARS-SPARC axis. As an extracellular matrix glycoprotein, SPARC modulates cell adhesion, migration and remodeling, and actively participates in processes such as bone formation, muscle regeneration, angiogenesis and tissue repair. Its affinity for calcium and collagen makes it a key player in the maintenance and remodeling of bone tissue, while in muscle it promotes myoblast differentiation and post-exercise metabolic balance. It also exerts metabolic and immunological functions, promoting glucose homeostasis, inhibiting inflammation (such as NLRP3 inflammasome activation) and contributing to adipose tissue regulation, with effects observed in animal models of obesity and insulin resistance [116,117,118,119,120].

From a therapeutic point of view, SPARC shows a high potential in metabolic, musculoskeletal and possibly oncological diseases. Strategies ranging from endogenous induction by physical exercise to exogenous administration as a recombinant protein or its incorporation in tissue engineering biomaterials to promote bone mineralization have been proposed. Studies also suggest an antitumor role of SPARC secreted by muscle, with protective effects against the development of colorectal cancer. Because of its ability to improve glucose tolerance, reduce fat mass, modulate the extracellular matrix and contribute to muscle regeneration, SPARC represents a promising therapeutic target to treat conditions such as osteopenia, sarcopenia, type 2 diabetes, chronic inflammation and fibrosis. Although less studied than other myokines such as irisin or IL-6, its versatile biological profile positions it as a key molecule in the field of exercise-associated metabolic and regenerative medicine [117,161,162].

### 4.10. Leukemia Inhibitory Factor

Leukemia inhibitory factor (LIF) is a pleiotropic cytokine belonging to the IL-6 family that is also classified as a myokine, since it is produced by skeletal muscle in response to muscle contraction, especially after endurance exercise or tissue damage [121]. Its expression is transiently regulated, and its secretion appears to be mainly local, acting in an autocrine or paracrine mode, since its levels are not usually detected in plasma. At the molecular level, LIF exerts its effects through the LIFR/gp130 receptor complex, activating JAK/STAT3, MAPK and PI3K/AKT signaling pathways. In muscle, LIF has essential functions: it stimulates satellite cell proliferation, regulates muscle regeneration, and enhances glucose uptake, highlighting its role not only in muscle repair but also in muscle metabolism [121,122,123,124]. As a myokine, LIF represents a molecular bridge between physical exercise and tissue regeneration, with promising potential therapeutic applications in regenerative and metabolic medicine.

### 4.11. Fibroblast Growth Factor 21

FGF-21 is a 181 amino acid protein classified as a hepatokine, but it also acts as a conditional myokine, as it can be secreted by skeletal muscle in response to stimuli such as prolonged exercise, fasting or mitochondrial stress [163,164]. Its expression in muscle is regulated by the PGC-1α pathway, as are other myokines such as irisin. Although its main organ of production is the liver, it is also expressed in adipose tissue and muscle, exerting endocrine, autocrine and paracrine effects. It acts through the FGF-R1c/3c receptors together with the β-Klotho cofactor, present in metabolically active tissues. FGF-21 improves glucose uptake (independently of insulin), reduces glucagon secretion and protects pancreatic function [125].

Functionally, FGF-21 regulates energy metabolism by increasing lipid oxidation, favoring ketogenesis, and promoting white adipose tissue browning in synergy with Metrnl and irisin. In addition, it has cardioprotective properties, reducing hypertrophy and improving cardiac function in contexts of metabolic stress. Its circulating levels are elevated in conditions such as obesity, type 2 diabetes, metabolic syndrome and cardiovascular disease, so it is also investigated as a biomarker of insulin resistance. Currently, FGF-21 analogs are being developed for use in the treatment of metabolic diseases such as diabetes, NAFLD and heart failure. Although it is not a classical myokine, its role as a metabolic stress-induced exerkinin makes it relevant in the muscle–metabolism axis and justifies its inclusion in reviews on myokines and exercise [165,166,167].

## 5. Towards a Functional Framework for Endocrine–Muscle Integration

From a synthetic perspective, the interaction between classical hormones and muscle-derived myokines can be reframed within a functional typology that distinguishes three levels of endocrine–muscle crosstalk: (1) external endocrine regulation, where systemic hormones (e.g., GH, IGF-1, testosterone, insulin) modulate muscle growth, metabolism and regeneration; (2) internal endocrine signaling, where myokines act locally in autocrine and paracrine loops to regulate muscle adaptation, inflammation and remodeling; and (3) muscle-to-organ communication, where myokines exert endocrine effects on distant tissues such as adipose tissue, liver, pancreas or brain.

This tripartite framework emphasizes the dynamic reciprocity between muscle and the endocrine system, positioning skeletal muscle not merely as a passive target, but as an active endocrine integrator that senses, responds to, and modulates systemic metabolic demands. Future research should explore how this regulatory network adapts to physiological transitions (e.g., exercise, aging) and pathological conditions (e.g., sarcopenia, endocrine myopathies), potentially identifying key nodal pathways that could serve as therapeutic targets.

## 6. Conclusions

In conclusion, the dual function of skeletal muscle—as both a target and a source of endocrine signals—has profound implications for the pathophysiology and treatment of endocrine–metabolic diseases. It contributes significantly to the regulation of metabolism, energy homeostasis and interorgan communication, impacting key organs such as the liver, adipose tissue, immune system and cardiovascular system. Muscle endocrine dysfunction, caused by hormonal resistance, chronic inflammation or physical inactivity, plays a central role in the pathophysiology of metabolic diseases such as obesity, type 2 diabetes and sarcopenia, as well as aggravating the prognosis in complex endocrine diseases. Thus, maintaining or improving muscle function through structured exercise, personalized hormonal therapies or anabolic agents represents a promising therapeutic strategy to prevent and treat metabolic–endocrine disorders. Advances in the understanding of myokines open new avenues for the development of diagnostic biomarkers and pharmacological targets to optimize metabolic and endocrine health in clinical practice. However, several challenges remain that limit the translational application of myokines. One of the most pressing is the lack of standardized methodologies for quantifying circulating myokines, both in terms of assay specificity and pre-analytical variability, which hampers comparability across studies and limits clinical applicability. Equally important is the incomplete identification and validation of specific receptors for many myokines—such as irisin or myonectin—which delays the understanding of their target specificity and downstream signaling pathways. Furthermore, the dynamic regulation of myokines by exercise or nutritional states is likely disease-specific. For example, IL-6 exhibits beneficial anti-inflammatory effects in insulin-sensitive muscle but may exacerbate inflammation in insulin-resistant states. This suggests that context-dependent expression and function must be addressed through detailed phenotyping in diverse clinical populations, including aging, sarcopenia, and endocrine myopathies.

Future research should aim to (1) develop validated and reproducible assays for key myokines and their isoforms, (2) elucidate receptor expression patterns and downstream effectors across tissues, and (3) define disease-specific myokine–hormone interaction profiles. These advances are essential to move toward the use of myokines as reliable biomarkers or therapeutic targets in endocrine–metabolic disorders.

## Figures and Tables

**Table 1:** Effects of hormones on skeletal muscle.

Hormone	Action	Main Effect on Skeletal Muscle
GH/IGF-1	Anabolic	They stimulate protein synthesis, satellite cell proliferation and muscle hypertrophy [12,13,14]
Testosterone	Anabolic	It promotes protein synthesis, inhibits protein degradation, activates satellite cells and inhibits myostatin [1,2]
Insulin	Anabolic	Increases glucose uptake, promotes protein synthesis and reduces protein degradation [1,8]
Glucocorticoids	Catabolic	They inhibit protein synthesis, induce insulin/IGF-1 resistance and activate degradation via ubiquitin–proteasome pathways [15,16]
Catecholamines	Catabolic (contextual)	They stimulate proteolysis, although in certain contexts (exercise) they can activate anabolic pathways [5,17,18].
Estrogens	Mixed	Improves mitochondrial metabolism and muscle regeneration; deficiency aggravates sarcopenia [19,20,21,22]
Adipokines (leptin, adiponectin)	Mixed	They modulate insulin sensitivity, inflammation and muscle mass; leptin may be deleterious in obesity [23,24,25]
Thyroid hormones (T3, T4)	Mixed	They stimulate mitochondrial biogenesis and protein synthesis [26,27,28]; hypothyroidism → weakness, hyperthyroidism → atrophy

Abbreviations: GH, growth hormone; IGF-1, insulin-like growth factor 1; T3, triiodothyronine; and T4, thyroxine.

**Table 2:** Effects of myokines on muscle and metabolism.

Myokine	Main Source	Main Effects
Irisin (derived from FNDC5)	Muscle during exercise	Induces browning of adipose tissue, improves insulin sensitivity, and promotes mitochondrial biogenesis [98,99]
Muscle IL-6	Muscle fibers	Stimulates lipolysis and fatty acid oxidation, modulates inflammation (anti-TNF-α), improves glucose uptake [100,101]
IL-15	Muscle fibers	Promotes protein synthesis, inhibits muscle apoptosis, improves lipid and glycemic metabolism [102,103,104,105,106]
Muscle BDNF	Muscle and CNS	Improves regeneration and oxidative metabolism, promotes neuroprotection and glycemic regulation [107,108]
Myonectin (CTRP15)	Muscle	Promotes fatty acid uptake, reduces circulating lipids, improves insulin sensitivity [109,110,111,112]
Myostatin	Muscle	Inhibits muscle growth, induces atrophy, activates catabolic pathways (SMAD2/3, NF-κB) [29,113]
Metrnl	Muscle, adipose tissue	Induces browning, improves insulin sensitivity, promotes muscle regeneration and angiogenesis [114,115]
SPARC	Muscle, bone	Promotes myoblast differentiation, modulates inflammation and glycemic metabolism [116,117,118,119,120]
LIF	Muscle	Stimulates satellite cell proliferation, promotes regeneration and glucose uptake [121,122,123,124]
FGF-21	Liver, muscle	It improves glucose uptake, promotes lipid oxidation and has a cardioprotective effect [125]

Abbreviations: BDNF, brain-derived neurotrophic factor; CTRP15, C1q/TNF-related protein 15; FGF-21, fibroblast growth factor 21; IL-6, Interleukin 6; IL-15, Interleukin 15; Metrnl, Meteorin-like; NF-κB, nuclear factor kappa B; FNDC5, fibronectin type III domain-containing 5; LIF, leukemia inhibitory factor; SMAD, SMA- and MAD-related protein; SPARC, secreted protein acidic and rich in cysteine; and TNF-α, tumor necrosis factor alpha.

## Data Availability

No original data are associated with this manuscript.
